# A Review of Translational Behavioral Assays in Depression Research

**DOI:** 10.3390/biology15090667

**Published:** 2026-04-23

**Authors:** Ayush Sabherwal, Julianna E. Peña, Anthony T. Lopez, Frederick L. Hitti

**Affiliations:** 1 Department of Neurological Surgery, University of Texas Southwestern Medical Center, Dallas, TX 75390, USA; ayush.sabherwal@utsouthwestern.edu (A.S.); julianna.pena@utsouthwestern.edu (J.E.P.); 2 Department of Neuroscience, University of Texas Southwestern Medical Center, Dallas, TX 75390, USA; anthony.lopez@utsouthwestern.edu; 3 Department of Psychiatry, University of Texas Southwestern Medical Center, Dallas, TX 75390, USA

**Keywords:** depression, anhedonia, rodents, humans, cross-species tasks, translational behavior, animal models, reward learning, effort motivation, cognitive bias

## Abstract

Major depressive disorder is a highly prevalent psychiatric disease, and many patients remain symptomatic despite treatment. Rodent models are central to preclinical depression research, but their translational impact is often constrained by the lack of direct human analogs. We conducted a review to identify behavioral tasks with direct cross-species analogs between rodents and humans. We focused on tests with comparable assay structures that measure depressive symptoms and related constructs. We followed PRISMA (Preferred Reporting Items for Systematic Reviews and Meta-Analyses) guidelines and included all relevant studies. After screening 9680 studies and applying inclusion and exclusion criteria, 62 studies were included. We identified 10 behavioral tests with comparable human and rodent versions. These tests include assays of positive and negative valence systems, affective bias, and cognitive systems. These cross-species behavioral tests help close the gap between animal and human paradigms and advance the understanding of the neurobiology underlying depression. Each test has distinct strengths and limitations in its implementation across species. These assays offer a promising bridge between human and rodent research, and continued efforts to standardize and develop these tests will help maximize their utility in enhancing the understanding of depression and developing more effective treatments for mood disorders.

## Simple Summary

Depression is a common and disabling illness, and unfortunately many people continue to experience symptoms even after receiving treatment. Scientists frequently study depression through behavioral tests in rodents to better understand the biological underpinnings of the disease. The structures of many rodent tests, however, do not resemble the tests used to study depression in humans. This limitation makes it difficult to translate findings from the rodent models to human disease. In this review, we conducted a broad literature search to identify behavioral tests used in both humans and rodents with comparable structures and methods. We identified 10 tasks that can be directly translated across species. These tasks measure key features of depression, such as changes in reward processing, emotional bias, and decision-making under uncertain conditions. These cross-species tests strengthen the connection between animal and human research. We also highlight areas for improvement to maximize the value of these models to ultimately develop more effective treatments for depression.

## 1. Introduction

Major depressive disorder (MDD) is a highly prevalent psychiatric illness, and many patients remain significantly symptomatic despite treatment [1,2]. Currently available interventions include psychotherapy, pharmacotherapy, and neuromodulatory approaches such as electroconvulsive therapy (ECT) [3,4,5,6]. Although these treatments can be effective, a substantial proportion of individuals fail to achieve full remission, and many continue to experience chronic functional impairment. MDD is thought to arise from a combination of genetic predisposition, neurobiological alterations, and environmental factors, which together contribute to the heterogeneity of the disorder and its varying treatment response. This ongoing disease burden underscores the need for a deeper understanding of the neurobiology of depression and for improved strategies to develop and test novel therapies.

Rodent models have been central to preclinical depression research and have contributed important insights into how stress, neurobiological systems, and candidate treatments influence behavior. Early work relied heavily on a relatively small set of behavioral tests that are sensitive to acute antidepressant treatment in unstressed animals, such as the forced swim test, tail suspension test, and sucrose consumption or preference tests [7,8,9,10,11,12]. These paradigms have been extremely prevalent, in part because they are easy to implement and reliably detect the behavioral effects of psychiatric drugs. However, they also highlight a key disconnect: clinical antidepressant trials require chronic treatment and target a complex, multi-symptom syndrome, whereas these traditional preclinical tasks capture only narrow behavioral readouts with limited resemblance to human assays.

Stress is a major risk factor for MDD, and extensive literature has leveraged chronic stress paradigms (such as chronic social defeat stress or chronic mild stress) to induce depression-relevant behavioral changes in rodents, including social withdrawal, helplessness, and reduced reward responsiveness [13,14,15,16]. These models have been particularly useful for dissecting stress susceptibility versus resilience by comparing animals that do or do not develop behavioral alterations after stress exposure [17]. Such designs mirror the human condition, in which only a subset of stressed individuals develop clinical depression [18]. The ultimate goal is to link individual differences in stress responses to underlying genetic, molecular, and circuit-level mechanisms.

Despite these advances, the translational impact of preclinical depression research has been constrained by a persistent gap between how behavior is measured in animals versus humans. Many of the most commonly used rodent tasks have no direct human analog. For example, humans are not evaluated in water tanks for “immobility” and clinical anhedonia is rarely quantified via unflavored sucrose intake. In contrast, human experimental psychology has developed a broad array of laboratory paradigms that measure specific dimensions of depression (e.g., reward responsiveness, effort-based decision-making, negative cognitive and affective biases, sustained attention, and cognitive flexibility) using well-controlled tasks and computational models [19,20]. Closing the gap between animal and human paradigms requires behavioral assays that are structurally similar across species and investigate comparable psychological constructs.

Animal models cannot be used to assay human mental experiences such as guilt, worthlessness, or suicidal ideation. The goal of preclinical work is therefore not to model “depression,” but to capture measurable behavioral and biological features that correspond to specific symptom dimensions or intermediate constructs. From this perspective, the key question is not whether an animal “has depression,” but whether a task can reliably index processes like reward learning, effort valuation, negative bias under ambiguity, or cognitive control in a way that is meaningfully comparable to human performance [21].

Several groups have now designed rodent tasks that mirror human experimental paradigms used in depression research [22,23]. These directly translatable tasks are typically matched across species in terms of stimuli (e.g., visual or auditory cues), trial structure, probabilistic contingencies, and primary outcome measures (e.g., response bias, win-stay/lose-shift behavior, effortful choice, accuracy, and reaction times).

With these considerations in mind, we undertook a review to identify and outline the behavioral tasks that have been implemented in both humans and rodents with comparable assays. In particular, we took note of the tasks that have been used to probe depressive symptoms or closely related constructs. Our goal was to describe the advantages and limitations of these directly translatable paradigms, to highlight areas of convergence and divergence between species, and to outline priorities for future work aimed at optimizing cross-species behavioral assays for depression research.

## 2. Materials and Methods

### 2.1. Search Strategy

We used the PubMed and Web of Science databases to identify studies employing rodent behavioral tests that have human analogs. We specifically sought tests that assay symptom domains relevant to depression, including anhedonia, sleep disturbance, psychomotor impairment, and cognitive dysfunction. The following search strategy was used to identify studies for inclusion in this review: (“rodent” OR “rat” OR “mouse”) AND (“human”) AND (“anhedonia” OR “sleep depression” OR “psychomotor depression” OR “cognitive depression”). All searches were conducted in “All Fields.” The search terms were intentionally broad to ensure all relevant articles would be captured. No publication date limits were applied. The initial search was performed on 21 February 2025.

Records were exported to Covidence [24] for deduplication, title/abstract screening, and full-text review. This review followed PRISMA (Preferred Reporting Items for Systematic Reviews and Meta-Analyses) guidelines and the PRISMA-ScR extension for reviews [25].

There is an inherent risk of publication bias (e.g., preferential publication of positive results) as well as potential bias due to variable study quality. Given that our primary goal was to provide a comprehensive overview of directly translatable behavioral tasks rather than to perform a quantitative meta-analysis, we followed PRISMA-ScR guidelines and did not conduct a formal risk-of-bias or study quality assessment. Instead, we summarize major design features and highlight limitations qualitatively.

### 2.2. Study Inclusion and Exclusion Criteria

All studies retrieved from the database search were first screened by title and abstract to determine relevance. Studies were eligible for a full-text review if they met the following criteria:

1. Reported original empirical data;
2. Included a behavioral task with a human and rodent version that has been used to study depressive symptoms, mood disorders, or closely related constructs (e.g., anhedonia, reward learning, negative bias, cognitive control);
3. Assessed behavior in the context of depression, depressive symptoms, or depression-relevant manipulations (e.g., chronic stress, anhedonia models, pharmacologic interventions, or clinical MDD samples).

We excluded review articles, commentaries, methodological notes without behavioral data, purely human or purely rodent studies without a cross-species analog, and studies in languages other than English. Reference lists of included articles and related reviews were examined to identify additional relevant studies not captured by the initial database search. Any additional eligible studies identified through reference screening were added to Covidence and processed with the same inclusion/exclusion criteria.

### 2.3. Data Extraction and Synthesis

For each included study, we extracted the following information where available: species and sample characteristics, human task variant, rodent task variant, primary behavioral outcomes (e.g., accuracy, response bias, win-stay/lose-shift, effort choices, reaction time, lick responses), depression-related group or manipulation (e.g., MDD diagnosis, chronic stress, pharmacologic treatment), and key findings relevant to depressive symptoms or constructs.

Given the heterogeneity of the included studies, we did not attempt a quantitative synthesis. Instead, we organized tasks into conceptual groups based on the target construct and task structure and provided a narrative synthesis of each paradigm.

## 3. Results

**Figure 1:**
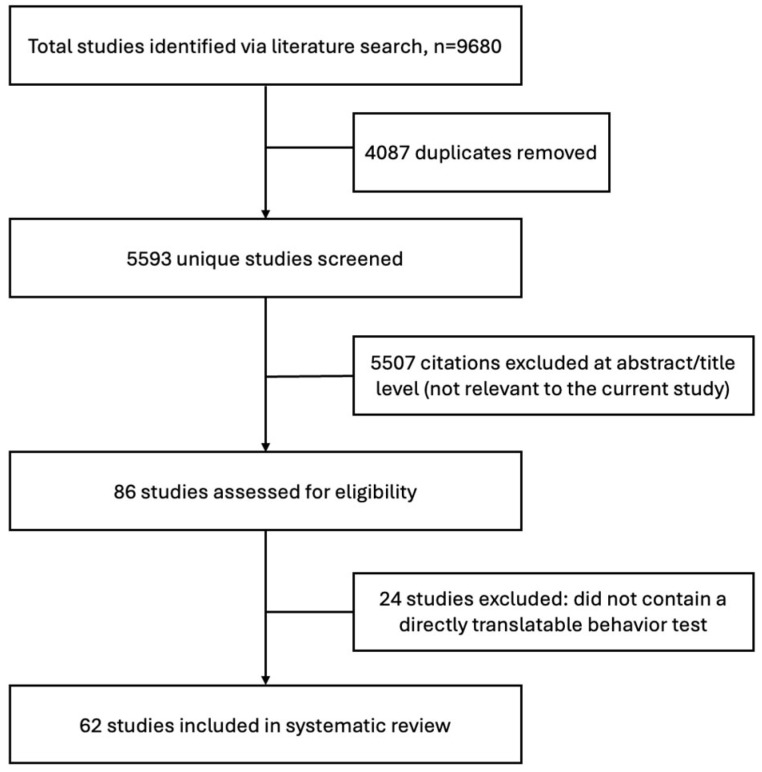
PRISMA flowchart of studies screened and selected for inclusion in the review.

Our initial search yielded 9680 studies. A total of 4087 duplicate references were removed, leaving 5593 unique records for title and abstract screening. Of these, 86 full-text studies were assessed for eligibility; 24 were excluded because they did not include a directly translatable behavioral test (e.g., studies employing rodent-only paradigms such as the forced swim test or sucrose preference test that lack a direct human analog). The final sample comprised 62 studies that met all inclusion criteria (Figure 1).

From the included studies, we identified 10 behavioral tests that have both human and rodent versions with comparable structure. These tests were the Judgment Bias Test (JBT), Continuous Performance Test (CPT), Probabilistic Reward Test (PRT), Probabilistic Reversal Learning (PRL), Effort-Expenditure for Rewards Task (EEfRT) and related rodent effort-based choice tasks, Paired Associates Learning (PAL), the Cognitive Effort Motivation Task (CEMT) and rodent Cognitive Effort Task (rCET), the Wisconsin Card Sorting Test (WCST) and two-choice rule-switch task, the Sweet Taste Test (STT) and brief-access taste assays, and the Affective Bias Test (ABT). These tasks range from assays of positive valence systems (reward responsiveness, effort, taste hedonia), negative valence and affective bias, and cognitive systems (attention, learning and memory, cognitive control).

**Figure 2:**
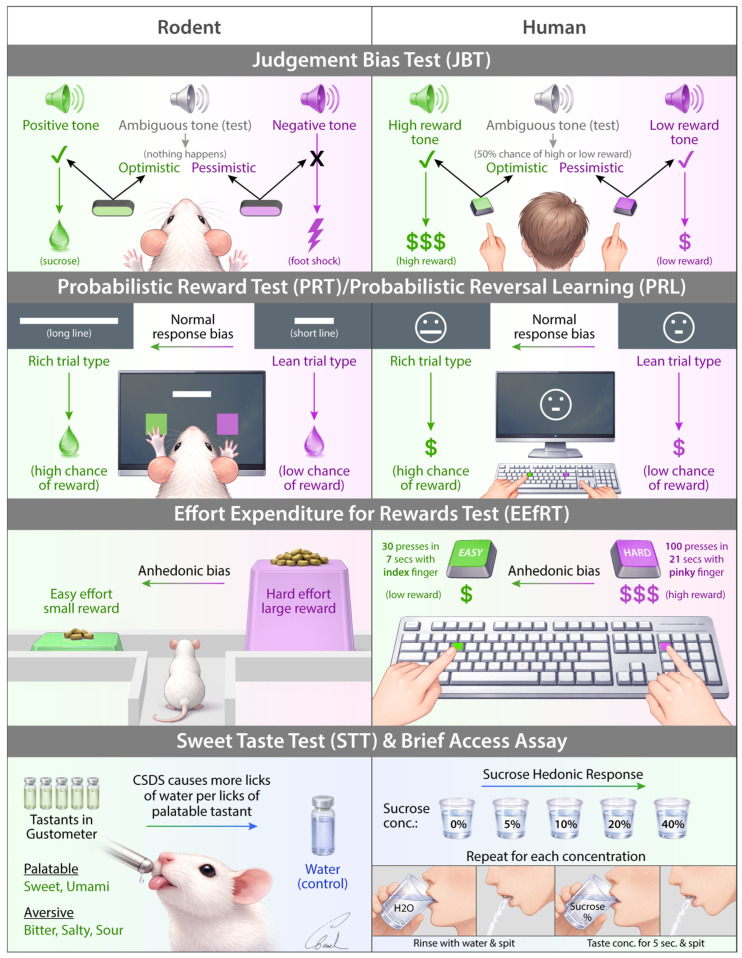
Illustration of several rodent and human cross-species behavioral tests relevant for depression research. CSDS (Chronic Social Defeat Stress), conc. (concentration). Note that this figure does not establish strict cross-species equivalence.

**Figure 3:**
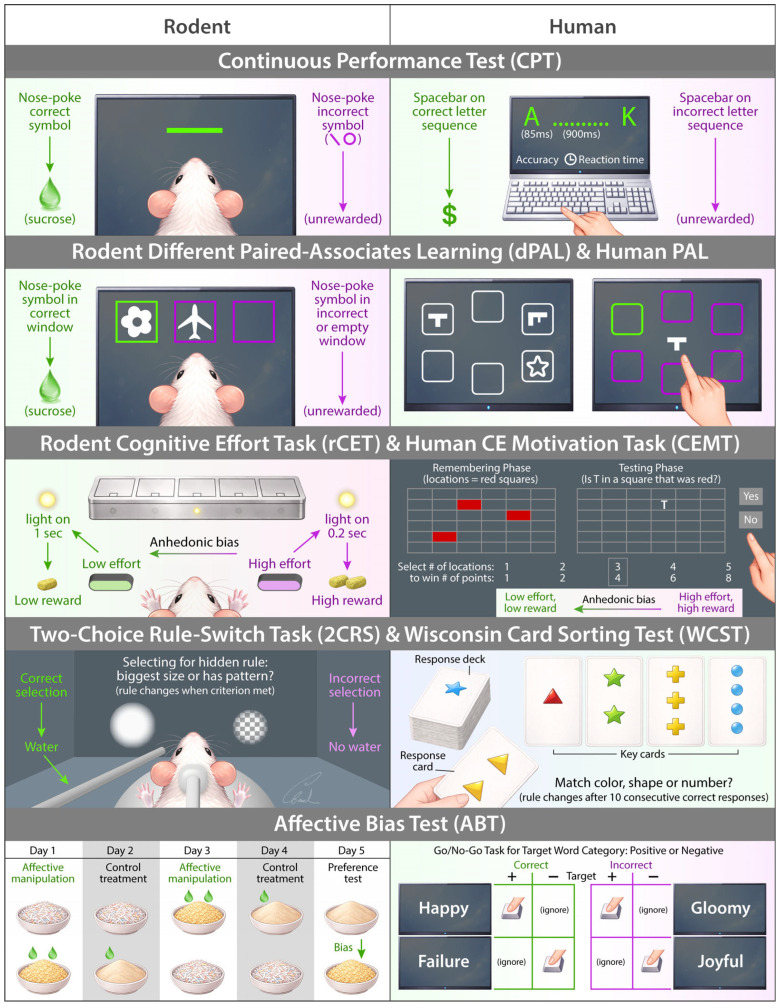
Additional illustration of several rodent and human cross-species behavioral tests relevant to depression research. Note that this figure does not establish strict cross-species equivalence.

These behavioral tasks are detailed below and summarized in Tables S1 and S2. The most salient tests are illustrated in Figure 2 and Figure 3.

### 3.1. Judgment Bias Test (JBT)

Several studies employed the Judgment Bias Test (JBT)—also called cognitive bias testing or ambiguous-cue interpretation—to measure how affective state shifts the interpretation of ambiguity in rodents [26,27]. In the version of the task employed in humans, subjects first learn two reference tones (1000 Hz and 500 Hz) that are predictive of distinct outcomes (larger vs. smaller monetary rewards, or monetary reward vs. avoidance of an aversive sound) [28,29]. Following the acquisition, ambiguous probe tones at intermediate frequencies are played and reinforced probabilistically. A reduced tendency to classify the probe tone with the better outcome (or increased avoidance) indicates a negative bias. Ambiguous tone trials consistently resulted in higher reaction times (RT) than the acquisition trials. In the affective tone discrimination task [28], at the most ambiguous tone (closest to the 750 Hz midpoint), subjects were more likely to avoid the aversive sound than to pick the monetary reward; this avoidance bias was significantly correlated with anxiety (VAS, STAI-State after controlling for BDI/PHQ-9 for depressed mood). In Ref. [29]’s high vs. low reward version: symptomatic subjects (a mixed group of subjects that met criteria for mood or anxiety disorders) chose the high-reward response less often on the mid-tone and showed a lower drift rate (the amount of prior evidence required to classify a tone as high reward) towards the high-reward boundary.

Given the ability of the JBT to measure negative biases under uncertainty, several groups translated the task for use in rodents using either spatial or auditory variants [26,27]. In the spatial version, mice are trained on a radial arm maze where one arm leads to a positive outcome (access to the home cage) and another arm leads to a negative outcome (air puff). Ambiguous probe arms are positioned centrally or near the positive/negative reference arms. In probe tests, approach latencies varied with spatial ambiguity: near-positive arms were reached fastest, then the central arm, and the near-negative arms slowest. The central arm elicited the highest ambiguity. Mice trained only on positive outcomes approached the central probe faster and spent longer investigating the target hole than mice trained on negative outcomes, suggesting a bias in the interpretation of an unfamiliar location.

In the auditory version of the task [27], mildly food-restricted rats are trained using operant boxes to associate one tone with a reward. The rat must press the “positive” lever to receive a sucrose solution. Another tone signals punishment, and the rats must press the “negative” lever to avoid an electric shock. Following training, a mid-tone is added between the frequencies; lever pressing during the tone terminates the tone but is not reinforced. After a three-week chronic social stress paradigm (daily resident-intruder confrontation), the rats’ choices shifted towards pressing the negative lever more often when the ambiguous tone was presented, reflecting a “pessimistic” negative bias.

### 3.2. Continuous Performance Test (CPT)

Several studies employed the continuous performance test (CPT) to measure sustained attention in rodents and humans [30,31]. In one version of the task employed in humans (Vigil CPT), a random letter flashes on a screen for 85 ms, followed by 900 ms of interstimulus interval. Subjects are instructed to respond only to the target sequence of an “A” followed by a “K” by pressing the spacebar, and accuracy and reaction time are measured. Although accuracy was similar across groups, patients with major depression or bipolar depression (DSM-IV) showed significantly higher reaction time variability than controls [31].

Given the ability of the CPT to measure sustained attention, Ding et al. [30] sought to translate this task for use in rodents. Mildly food-restricted rats in touchscreen chambers are trained to nose-poke to a single rewarded stimulus (horizontal or vertical bars) for a sucrose reward, while withholding responses to four unrewarded stimuli (e.g., diagonal lines or a circle pattern). Hits, misses, false alarms (nose-poking to a non-signal), correct rejections (withholding responses to the incorrect stimuli), reaction times, and signal-detection metrics were measured. Administration of drugs with stimulant-like properties (methylphenidate, ABT-594, and modafinil) increased hit rate, false alarm rate, response bias, and blank touches, while donepezil and memantine (drugs prescribed for dementia) decreased the same measures. Ding et al. [30] argue that because rats must discriminate sequentially presented, visually patterned target and non-target stimuli at a single location (seen in standard human CPTs), the touchscreen rodent Continuous Performance Test (rCPT) provides a close analog of human attentional performance.

### 3.3. Probabilistic Reward Test (PRT)

Several studies employed the Probabilistic Reward Test (PRT) [32,33,34,35,36] to measure reward responsiveness and learning in rodents and nonhuman primates. In the version of the task employed in humans, subjects are required to discriminate between two similar visual stimuli (i.e., a long mouth vs. a short mouth on a cartoon face) [37]. Subjects only receive feedback when they correctly discriminate between the two stimuli, but feedback is given more frequently for the “rich” stimulus compared to the “lean” stimulus (75% versus 25% of the time, respectively). The rich stimulus is thereby rewarded more frequently, and over repeated trials, participants tend to bias their responses toward the rich stimulus. This probabilistic design allows experimenters to quantify reward responsiveness. The key behavioral outcome is response bias, which measures an individual’s sensitivity to subtle differences in reinforcement probability. Importantly, individuals with MDD show blunted response bias compared to controls, suggesting a reduced hedonic capacity in these individuals [38].

Given the PRT’s ability to measure reduced reward responsiveness in MDD patients, several groups have translated this test for use in rodents and nonhuman primates [32,33,34,35,36]. In these studies, subjects were trained in operant chambers with (rodent) or without (nonhuman primate) mild food restriction to discriminate between visual stimuli presented via touchscreen (lines of differing lengths) or auditory stimuli (tones varying in duration). In rodents and primates, correct choices were reinforced with food rewards (e.g., sucrose pellets or sweetened condensed milk), whereas in the human version, reinforcement is typically monetary or symbolic. Both rodents and nonhuman primates acquired the task (>80% correct response rate) and showed a response bias, measured as log b, towards the more frequently rewarded stimulus. Log b measures response bias using the relative number of correct responses on rich versus lean trials. Values above zero indicate a bias toward the rich stimulus, and larger values reflect a stronger bias. Investigators measured rodent/nonhuman primate response bias under varying reward contingencies to generate an average response bias that was similar to that seen in humans. Most studies found that a 60%:20% rich:lean reward ratio generated log b values similar to those observed in human studies (~0.2–0.3).

### 3.4. Probabilistic Reversal Learning (PRL)

Several studies employed the Probabilistic Reversal Learning (PRL) task [39,40,41] to measure cognitive flexibility and reward feedback sensitivity in rodents. In the version of the task employed in humans, subjects make a selection between two visual stimuli (e.g., a set of red bars vs. a set of green bars) [42]. The correct stimulus is rewarded more frequently (80% of the time) than the incorrect stimulus (20% of the time). After a defined number of trials, the correct and incorrect stimuli are swapped to measure reversal learning (cognitive flexibility). Similar to the PRT, the probabilistic design of the PRL allows the experimenter to measure a subject’s reward feedback sensitivity. Since some incorrect choices are rewarded and some correct choices are punished, win-stay and lose-shift behavioral decisions can be quantified. The percentage of trials in which the participant shifts to the incorrect stimulus when the correct stimulus is punished (lose-shift) is quantified and termed the negative feedback sensitivity (NFS). Interestingly, patients with major depressive disorder (MDD) are unimpaired in task acquisition and reversal learning, but these patients do exhibit significantly greater NFS compared to controls [43].

Given the ability of the PRL task to assay reward feedback sensitivity, which is selectively impaired in patients with MDD, several groups sought to translate this task for use in rodents [39,40,41]. All of these studies used operant chambers to train food-restricted rodents to select between two visual stimuli presented either via a touchscreen [40,41] or an illuminated hole [39]. In the human studies, the reward was either symbolic or monetary. In the rodent version of the task, food/sucrose pellets were used as a reward. The rodent studies demonstrated that both mice and rats are able to acquire the task; notable behavioral differences were found between rodents and humans. Of particular interest, there was a large difference in NFS between rodents and humans [40]. In the human version of the PRL, the correct choice is rewarded 80% of the time. The two rat studies used this same reward probability [39], while the mouse study varied the reward probability to investigate how mouse behavior changed relative to the probability of obtaining a reward when the correct stimulus was chosen. Ineichen and colleagues [40] found that when the correct choice was rewarded 80% of the time, mice exhibited high NFS (the correct stimulus was selected near chance levels). This was similar to the NFS found in rats (50–60%) [39,41]. In contrast, when the correct stimulus is rewarded 80% of the time in humans, NFS is much lower (10% in controls vs. 30% in patients with MDD). Ineichen and colleagues [40] found that increasing the reward probability of the correct stimulus to 90% decreased the NFS as low as 40% in some mouse strains.

### 3.5. Effort-Expenditure for Rewards Test (EEfRT)/Joystick-Operated Runway Task (JORT) and Rodent Effort-Based Choice

Several studies employed the Effort-Expenditure for Rewards Task (EEfRT) and other similar assays [44,45,46,47,48] to measure effort-based reward motivation and cost–benefit decision-making. In one version of the task employed in humans, subjects choose between a low effort/low reward option (e.g., 30 button presses within 7 s using the dominant index finger to win $1.00) and a high effort/high reward option (e.g., 100 presses within 21 s using the non-dominant little finger to win $1.24–$4.30) [49,50]. Trials varied in reward magnitude and win probability (e.g., 12%, 50%, 88%), which were displayed to the subject before choosing. As reward size and probability vary across trials, the proportion of high-effort choices reflects a subject’s willingness to work for a reward under uncertain conditions. Subjects with elevated trait and state anhedonia (Chapman Physical and Social Anhedonia Scales, Beck Depression Inventory, etc.) exhibited a reduced tendency to select the high effort/high reward option, indicating diminished motivation and reward valuation.

Slaney et al. [49] also evaluated the ability of the Joystick-Operated Runway Task (JORT) to measure differences in reward motivation between individuals with high and low anhedonia scores. They found no difference in effort expenditure (force exerted on the joystick) between the two groups in this task, signifying that the EEfRT task may be a more useful tool.

Given the ability of the EEfRT to quantify effort-based motivation, which is impaired in individuals displaying depressive traits, several groups sought to translate this task for use in rodents [44,45,46,47,48]. One assay to measure effort-related choice is the food-choice test, in which rodents under mild food restriction are trained to press a lever to earn a preferred reinforcer (food reward pellets) on a fixed-ratio (e.g., FR5) schedule. Standard chow is freely available during the test, so the subjects have a choice between expending more energy (5 lever presses) for a preferred food versus expending less energy to consume the freely available standard chow. Under baseline conditions, rats predominantly lever-press for the preferred food pellets and eat little chow. After manipulations such as tetrabenazine administration (i.e., dopamine/monoamine depletion), rats lever-press less frequently and choose the low-effort lab chow more often, suggesting that the food-choice assay is sensitive to depression-related motivational deficits. Another variation in this test uses a touchscreen [47]. During this test, mice rear and press an elevated panel on an FR1 schedule to obtain a strawberry milkshake, while standard pellets are freely available. Additionally, a progressive-ratio variant uses the same operant setup as the food-choice test but increases the lever pressing requirement within the session, resulting in a maximum (the highest ratio completed by the rat) that reflects willingness to expend increasing effort. Dopamine manipulations (e.g., administration of DA antagonists) lower maximum effort expended and quicken the switch to chow [44].

Another test to measure effort-related choice in rodents is the T-maze Barrier Choice Task [46]. In this paradigm, one arm offers high reinforcement density (4 × 45 mg Bioserve pellets) but requires climbing a 44 cm tall vertical barrier, whereas the other arm provides less reinforcement (2 × 45 mg pellets) with no barrier. Healthy, mildly food-restricted rats typically favor the high-payoff arm despite the effort required to obtain the reward. Manipulations such as haloperidol or dopamine depletion in the nucleus accumbens shift choice away from the high effort/high value arm, but only when the barrier is present. In all the aforementioned tests, a decreased willingness to exert effort is analogous to the psychomotor fatigue and reduced effort-based reward pursuit that many patients with MDD experience.

### 3.6. Paired Associates Learning (PAL)

Several studies used the Cambridge Paired Associates Learning (CANTAB-PAL) task to assess associative memory in patients with mood disorders [51,52,53,54]. In the version of the task employed in humans, subjects view patterns appearing sequentially in boxes at varying locations on a screen; the boxes are then covered and subjects are required to indicate in which box each pattern was initially presented [52,54]. The number of locations to be remembered increases across stages (e.g., 2, 3, 6, 8). Errors, trials to criterion, response latencies, and stages completed are measured. Mixed/manic bipolar and MDD subjects (DSM-IV) made significantly more errors than healthy controls. Following remission from MDD, PAL performance improved but remained below control levels.

Given the ability of the PAL to measure visuospatial associative memory, which is impaired in depressed patients, Martis et al. [53] sought to translate the task for use in rodents through the touchscreen different Paired-Associates Learning task (dPAL). In dPAL, three visual symbols are presented, with each one displayed in a specific window. On each trial, two of the symbols appear with one in the correct window and the other in an incorrect window (the remaining window is left blank). Mildly food-restricted rats are required to touch the symbol displayed in the correct window to receive a food reward. Accuracy, errors, trials to criterion, and response latencies are measured. Following chronic mild stress (CMS), rats required more trials to reach the criterion than non-stressed controls. Animals resilient to stress acquired the task at a similar rate to control animals. Thus, the dPAL is able to detect cognitive performance decline in animals susceptible to stress.

### 3.7. Cognitive Effort Motivation Task (CEMT)/Rodent Cognitive Effort Task (rCET)

Several studies used the rodent Cognitive Effort Task (rCET) to measure willingness to expend cognitive and physical effort for reward [55,56,57]. In the version of the task employed in humans (Cognitive Effort Motivation Task, or CEMT), subjects choose between remembering one location for one point (low-effort/low-reward) or 2–5 locations for 2–8 points (higher-effort/higher-reward) [58]. Subjects are then given 2.5 s to remember a square grid in which the chosen number of squares are highlighted in red. During the testing phase, a “T” is briefly presented on the grid and subjects indicate whether it fell on a previously red square. The test is repeated five times and points are awarded if at least 4 of the 5 trials are correct. Subjects also rated the cognitive and physical demand of each trial on a 0–10 scale. Although MDD patients and healthy controls had similar decision times and execution accuracy, patients with depression reported significantly higher cognitive (but not physical) demand across memory loads and chose the higher-effort choice less often across all reward and effort levels.

Given the CEMT’s ability to measure cognitive effort, several groups sought to translate this task for use in rodents [55,56,57]. The rCET is performed in five-hole operant chambers where rats choose the trial difficulty before each trial. Selecting the low-effort/low-reward (LR) lever triggers a stimulus light for one second and dispenses one pellet for a correct nose-poke. The high-effort/high-reward (HR) lever triggers a stimulus light for 0.2 s but yields two pellets for a correct nose-poke. Choice preference, errors, and reaction times are measured.

The rCET has been used to demonstrate that dopamine antagonism reduces willingness to choose high-effort options on physical tasks (EDT—see EEfRT) but not tasks with increased cognitive demand (rCET) [57]. Pharmacologic inactivation of the anterior cingulate cortex reduced high-effort choice and increased premature responses/omissions [55,56].

### 3.8. Wisconsin Card Sorting Test (WCST)/Two-Choice Rule-Switch Task

Several studies employed rule-switching paradigms to measure cognitive flexibility across species [59,60]. In the version of the task employed in humans, four key cards are displayed (one red triangle, two green stars, three yellow crosses, and four blue circles). On each trial, subjects sort a response card (e.g., three red circles) under a key card and are given correct/incorrect feedback while the sorting rule (color, shape, number) is hidden. After 10 consecutive correct sorts, the rule switches without warning. Categories completed, trials to criterion, and errors are measured. All controls completed all six categories, whereas only 82% of dysphoric subjects completed all categories. Furthermore, dysphoric subjects took significantly more trials than controls to carry out the task and made more preservative and non-preservative errors [60].

Given the ability of the WCST to measure cognitive flexibility and set-shifting, both impaired in dysphoria, Biró et al. [59] sought to translate this task for use in rodents. In the visual two-choice rule-switch task, head-fixed, water-restricted mice run on a ball in a virtual reality arena. Each trial presents two options that differ in size and pattern, and the mouse steers left/right to choose. Correct choices (according to the hidden rule “Pattern” or “Big”) are rewarded with water; incorrect choices are not rewarded. The rule switches within a session once the performance criterion is met, and trials to criterion and errors are measured. Following the rule change, mice showed an increased number of trials to criterion and slower responses on conflicting trials. Post-switch errors were primarily regressive (failure to maintain the new rule).

### 3.9. Sweet Taste Test (STT)

Several studies employed the Sweet-Taste Test (STT) and brief-access assays to measure sweet-taste detection thresholds in humans and rodents [61,62]. In the version of the task employed in humans, subjects taste solutions of increasing sucrose concentration (0–40%), keeping each solution in their mouths for five s before spitting and rinsing [61]. The sweet perception threshold is the lowest concentration at which the subject first perceives sweetness. Hedonic response is assessed by rating the pleasantness of five sucrose concentrations. Although hedonic responses were similar across groups, depressed patients (Montgomery-Asberg Depression Rating Scale) showed significantly higher sweet-taste perception thresholds than controls, as well as lower pleasure and higher displeasure scores. Across individuals, higher Physical Anhedonia (Chapman) was associated with decreased pleasantness.

In the rodent version of this task, brief-access tests are used to measure sweet-taste sensitivity after stress exposure [62]. Following 10 days of chronic social defeat stress, mice are tested on a brief-access gustometer (shutter open for 5 s) to sample a sucrose solution and other taste compounds representing the five basic tastes. Responses were quantified as licks to the tastant minus licks to water, and lick ratios for aversive taste solutions. Stressed mice showed significantly reduced sensitivity and response to sweet and umami tastes (minimal changes for salty, sour, or bitter).

### 3.10. Affective Bias Test (ABT)

Two studies employed the Affective Bias Test (ABT) [63,64] to evaluate how affective states influence reward learning and memory in rodents. In the version of the task employed in humans, subjects complete a go/no-go paradigm in which emotionally valenced words (i.e., happy, joyful, cheerful vs. sad, hopeless, failure, etc.) are presented one at a time as either a target or a distractor. Subjects are instructed to indicate the valence of the target category (i.e., positive or negative) and ignore the distractor words [65,66,67]. After a set number of trials, the rule is reversed and subjects must respond to the opposite emotional category. Task performance is measured through reaction times, errors (responses to distractor stimuli), and omissions (failure to respond to target stimuli). Patients with MDD show impaired performance when positive words are the target, reflecting a negative affective bias, whereas manic patients demonstrate the opposite pattern, showing a positive affective bias. Harfmann et al. [67] extended this design to suicidal populations, showing that depressed suicidal ideators with a history of suicide attempts made more commission errors to negative distractors and responded faster to negative words than positive words, suggesting a heightened mood-congruent bias.

Given the ability of the human affective bias paradigms to detect mood-congruent processing biases, several groups sought to translate this task for use in rodents [63,64]. In these studies, rats first learn to associate digging substrates (i.e., sawdust, paper, etc.) with a sucrose reward during four separate pairing sessions, two conducted under neutral conditions and two under an affective manipulation (i.e., drug administration, stress, etc.). For the choice test, both substrates are presented together, and the animal’s preference reveals the affective bias encoded during learning. When these learning sessions occur under acute antidepressant treatment, rats show a positive bias, preferentially choosing the substrate paired with the drug. In contrast, manipulations known to induce negative affect (FG7142, restraint stress, etc.) produce a negative bias, with animals avoiding the substrate paired with the adverse state.

To extend this approach to chronic treatments, a modified version of the task (mABT) was developed in which one substrate is paired with a higher value reward (i.e., two sucrose pellets) and the other with a lower value reward (i.e., one pellet). Under normal conditions, rats consistently bias toward the high-value option. Prolonged exposure to pro-depressant manipulations blunts the positive bias, while antidepressant treatments preserve or enhance it [64].

## 4. Discussion

Major depressive disorder is a disabling illness with a clinically diverse patient population, and many patients remain symptomatic despite receiving standard-of-care treatments. Animal models have been indispensable for investigating the neurobiology of stress, but a longstanding challenge has been the limited alignment between traditional rodent assays (e.g., forced swim and sucrose preference tests) and the cognitive and affective tasks used in human research and clinical trials. Many conventional rodent tests lack clear human analogs, making it difficult to directly compare behavior across species or to link preclinical findings to specific depressive symptom domains in patients.

In this review, we identified 44 studies that implemented 10 behavioral tasks with closely matched versions in humans and rodents. These assays span judgment and affective bias, sustained attention, probabilistic reward learning, effort-based decision-making, associative memory, cognitive flexibility, taste sensitivity, and feedback sensitivity. Across tasks, convergent evidence suggests that depressive symptoms (including anhedonia, reduced motivation, negative bias under uncertainty, and cognitive inflexibility) can be captured in analogous paradigms across species. At the same time, parameter choices (e.g., reward probability, effort cost, stimulus type) and outcome measures can differ substantially between studies and species, which has important implications for translational validity. Moreover, most rodent studies rely on a limited set of stress models (e.g., chronic mild stress, social defeat), and human samples have varied considerably in clinical severity, making it difficult to determine how well these tasks generalize across patient populations.

In the following sections, we discuss the strengths and limitations of each assay, consider issues of model validity in the cross-species context, and highlight future directions for developing a more comprehensive and mechanistically informed behavioral task battery for translational depression research.

### 4.1. Judgment Bias Test (JBT)

The Judgment Bias Test measures how affective state shapes the interpretation of ambiguity. Human versions of these tasks use ambiguous tones predicting reward versus punishment [28,29], while rodent versions rely on spatial or auditory discrimination with ambiguous probe cues [26,27]. Humans with depressed or anxious traits show a tendency to classify ambiguous stimuli as signaling worse outcomes, and stressed rodents show analogous “pessimistic” shifts in lever choice or approach latency.

There are several advantages to this task. First, the human and rodent versions are nearly directly analogous (particularly in the case of the auditory cue rodent version). Second, the cues can be manipulated easily and precisely. Third, the rodent version has been validated in a rodent stress model. A few limitations include the fact that the human version has not been validated in patients with depression and that the human and rodent rewards are different (monetary versus food, respectively).

### 4.2. Continuous Performance Test (CPT)

CPT paradigms measure sustained attention and vigilance, with human versions typically requiring rapid responses to rare target sequences and rodent versions using touchscreen or operant discrimination of signal versus non-signal stimuli [30,31]. Depressed patients show increased reaction time variability with preserved accuracy, suggesting attentional instability.

Strengths of the CPT include a highly similar task structure between rodent/human paradigms (rare targets embedded in frequent non-targets) and rich signal-detection metrics (hits, false alarms, bias). However, relatively few studies have applied CPT to stress-based rodent models of depression, and the link between CPT performance and core depressive symptoms (e.g., anhedonia, depressed mood) remains less direct than for reward-based tasks. In addition, sustained attention is more sensitive to general arousal in both species. As such, the CPT may be a useful complementary tool for characterizing cognitive deficits in translational depression research but likely needs to be embedded within broader behavioral batteries.

### 4.3. Probabilistic Reward Task (PRT)

The PRT is a prototypical translational assay of reward responsiveness and reinforcement learning [32,33,34,35,36,37]. Across species, subjects discriminate between subtly different stimuli, with asymmetric reinforcement favoring one option (“rich” stimulus) and response bias toward that option serving as the primary outcome measure. Human subjects with higher depressive or anhedonic symptomatology show blunted response bias [38].

A key strength of the PRT is its strong construct validity: it isolates sensitivity to relative reinforcement probabilities while controlling for overall accuracy. Furthermore, the cross-species variations in the task are directly analogous. Another advantage is that the task has been validated in animal stress models. One limitation of the PRT is the fact that rodents and primates often require different rich:lean reinforcement ratios compared to humans to show comparable biases, indicating species differences in sensitivity to probabilistic reinforcement. Despite this limitation, the PRT remains one of the clearest examples of a reward-learning assay that has been validated across species.

### 4.4. Probabilistic Reversal Learning (PRL)

The PRL is similar to the PRT, but instead of employing ambiguous stimuli as in the PRT, the PRL employs two distinct stimuli [39,40,41,42,43]. Additionally, the PRL adds a reversal learning component by swapping the correct and incorrect stimuli during the test. Advantages of the PRL are that it has been validated in patients with MDD and the cross-species paradigms are very similar [43]. Limitations of this task are that negative feedback sensitivity (the primary outcome measure) differs across species. An additional limitation is that the task has not been validated in rodent stress models. Finally, MDD patients are unimpaired in reversal learning, so there is no clear reason to include the reversal component in preclinical studies.

### 4.5. Effort-Expenditure for Rewards Task (EEfRT) and Related Rodent Effort-Based Choice Assays

The EEfRT and related tasks assess willingness to exert increased physical effort for greater rewards [44,45,46,47,48,49,50]. Advantages of these tasks include a methodology to assess motivation and reward valuation. Another advantage is that an increased number of button presses (human version) is analogous to an increased number of lever presses (some rodent versions). There are several limitations to these tasks, however. First, there are multiple different protocols for the rodent version of the task. Second, the human version of the task has not been validated in depression patients. Third, the task has not yet been validated in rodent stress models.

### 4.6. Paired Associates Learning (PAL) and dPAL

The CANTAB-PAL task (humans) [51,52,54] and touchscreen-based dPAL (rodents) [53] both measure visuospatial associative learning and memory, a domain that is frequently impaired in patients with mood disorders. Major strengths of the PAL/dPAL tests are their near-identical stimulus presentation (patterns displayed on a touchscreen) and outcome metrics (errors, stages completed, trials to criterion) across species. Another key advantage of these tests is their validation in patients with depression and in rodents subjected to stress. A minor limitation is that these tests do not assay core depressive symptoms (anhedonia/depressed mood).

### 4.7. Cognitive Effort Motivation Task (CEMT) and Rodent Cognitive Effort Task (rCET)

While the EEfRT and related tasks measure willingness to exert additional physical effort during a task, the CEMT and rCET measure willingness to expend increased cognitive effort during a task [55,56,57,58]. The advantages of this task are that the human version has been validated in patients with depression. One limitation is that the rodent version of the task has not been validated in a stress model. Another limitation is that the rodent and human versions of the task are not directly analogous. Cognitive load is increased in the rodent version by shortening the duration of the stimulus presentation, but in the human version, the cognitive load is increased by increasing the number of required stimuli to remember.

### 4.8. Wisconsin Card Sorting Test (WCST) and Two-Choice Rule-Switching

WCST-like tasks test cognitive flexibility and set-shifting, which are critical for adapting to rule changes [59,60]. While dysphoric participants make more errors in this task compared to controls, the task has not been validated in patients with depression. Additionally, it has not been validated in rodent stress models. Another limitation is that the rodent version utilizes a head-fixed paradigm that is less naturalistic. Furthermore, the stress of head fixation may interfere with or confound studies of affective behavior.

### 4.9. Sweet Taste Test (STT) and Brief-Access Assays

The STT measures sweet taste detection thresholds and hedonic ratings in humans, and sweet taste sensitivity in rodents [61,62]. Strengths of these assays include validation in patients with depression and in rodent stress models. The tests have good face and construct validity. A minor limitation is that the rodent version does not measure sweet taste detection threshold per se. Furthermore, the rodent version of the task requires a gustometer, which may not be available in many laboratories.

### 4.10. Affective Bias (ABT) and Mood-Congruent Processing Paradigms

In humans, the ABT is a go/no-go task using emotionally valenced words [65,66]. Rodent paradigms use substrate–reward associations to assay affective bias [63,64]. The advantages of these tests are that they have been validated in patients with MDD and in rodent stress models. A notable limitation is that the rodent version varies in task structure.

### 4.11. Behavioral Test Validity

Traditionally, animal models have been evaluated in terms of predictive, face, and construct validity. Predictive validity refers to the ability of the model to induce or reverse a behavioral state across species. For example, in the context of depression research, a model that features stress to induce a behavioral state and/or antidepressants to reverse that state is considered to have good predictive validity. While several of the behavioral paradigms described above were validated in stress models, not all were. Furthermore, while the responsiveness of animals to acute antidepressant treatment was assessed, studies did not assess the effect of chronic antidepressant administration. Hence, further validation of these tasks should be done to increase predictive validity.

Face validity considers the similarity between the signs or symptoms of a human disease state and measurable behavioral metrics in animal models. The behavioral tasks detailed here optimize face validity by attempting to use the same behavioral readout across species. The aforementioned tasks vary in the strength of their face validity. Tasks such as the JBT [26,27,28,29] and the PRT [32,33,34,35,36,37] utilize nearly identical stimuli and behavioral readouts across species, so their face validity is high. Other tasks, such as the CEMT [55,56,57,58], have relatively less face validity because there are slight (but potentially meaningful) differences between the tasks across species. In the case of the CEMT, cognitive load is increased in humans by increasing the number of stimuli, whereas in rodents, cognitive load is increased by decreasing the stimulus presentation time. Although not every task reviewed here was developed with identical cross-species stimuli or task structure, all of the tasks detailed here have enhanced face validity compared to classically utilized tasks for measuring affective states in rodents such as the forced swim test. A minor limitation of all of the tasks described above is that the rewards are different across species (i.e., monetary reward in humans vs. food reward in rodents). Additionally, species differences in sensory dominance (e.g., olfaction vs. vision) and higher cognitive capacities may shape task performance and complicate direct comparison. These cross-species differences are likely unavoidable, but the development of a task that utilized the same punishments/rewards across species would enhance validity even further.

### 4.12. Comparative Synthesis Across Tasks

The ten paradigms reviewed here vary in their translational validity. Reward-based assays such as the PRT and STT offer the strongest cross-species alignment, with near-identical readouts, validation in both MDD patients and rodent stress models, and a strong link to anhedonic symptoms. Judgment-related paradigms (JBT, ABT) offer very similar task structure and growing validation, but further work extending these tasks to clinical populations would strengthen their translational utility. Associative memory tasks (PAL/dPAL) are valuable due to their methodological similarity, but do not target core depressive symptom domains. Effort-based (EEfRT), cognitive-effort (CEMT/rCET), cognitive flexibility (WCST), and sustained attention (CPT) paradigms target analogous behavioral constructs across species but would greatly benefit from further validation in clinical populations and rodent stress models to improve their cross-species validity. Overall, reward-processing tasks currently provide the most robust translational framework, while the remaining paradigms represent valuable tools that can be used in conjunction with other assays and to capture additional domains with continued validation efforts.

### 4.13. Assessing Major Depressive Disorder Symptom Domains Across Species

Early preclinical depression research often referred to “animal models of depression”. Indeed, some studies continue to utilize this terminology. Since depression is a uniquely human disease, there has been a more recent effort (NIH RDoC and others) [21,68] to guide preclinical efforts towards modeling symptom domains instead of disease states such as depression. Clinical depression encompasses multiple symptom domains including anhedonia, depressed mood, weight changes, sleep disruption, fatigue, feelings of worthlessness/guilt, cognitive impairment, psychomotor changes, and suicidal ideation. These varied symptoms do not align neatly within a single behavioral assay. In this review, we aimed to identify all relevant tasks (see methods) that assayed one or more depression symptom domains across species.

Reward responsiveness (a construct within the positive valence system domain) is measured by PRT, EEfRT and related effort-based tests, ABT, JBT, and STT. Cognitive domains are tested by CPT (sustained attention), PAL/dPAL (associative memory), WCST/rule-switch and PRL (flexibility and feedback learning), and CEMT/rCET (cognitive effort). Negative affective bias and heightened sensitivity to negative outcomes are captured by JBT, ABT, and PRL’s NFS metrics.

Utilization of the behavioral tasks identified here will serve to link studies of neural circuits to behavioral domains that can be mapped across species. The overarching goal would be to enhance the translational potential of preclinical studies. Furthermore, since depression symptomatology varies across individuals, if symptom domains are better characterized, treatments could be more precisely tailored to individual patients. These efforts would be in line with frameworks such as the NIH’s Research Domain Criteria (RDoC).

## 5. Future Directions

Our review identified several promising behavioral tasks that assay behavioral domains relevant to depression across species. Future work has the potential to utilize these paradigms, but also has the potential to improve upon them or develop additional novel tasks.

One area for improvement is the standardization of task parameters and structure. For example, some of the tasks have multiple variations (e.g., different stimulus types). If possible, the variations could be compared with a focus on choosing the variation that most closely mimics the human version of the task. Although the initial goal should be to mirror the human version of the task in rodents as closely as possible, investigators should be mindful that some parameters may need to be adjusted when translating the task across species. For instance, in the PRT, reward ratios should be adjusted so that the outcome metric (response bias) matches between species. Future task development should also consider how species differences in sensory dominance and cognitive complexity may influence task performance, and design paradigms that minimize the impact of these differences on cross-species comparisons. Similar caveats exist for other tasks, such as the physical and cognitive effort tasks. Another important area for future work is to further validate the tasks. Tasks should be validated in patients with depression as well as in rodent stress models. Ideally, the task would also be validated in patients and animals after antidepressant treatment to maximize predictive validity.

The majority of the studies identified here were devoted to the development of a single behavioral task. If possible, studies would benefit from the implementation of a battery of behavioral tests so that more information could be extracted from each experiment and differences between individuals could be further explored.

Relatively few studies identified here examined sex differences or age, despite strong evidence that depression risk and presentation differ by sex and across the lifespan. Studying both males and females and subjects of different ages would make these translational tools more representative of clinical populations. This line of work has the potential to identify and enable investigation of behavioral and/or circuit-level differences between patient populations.

Finally, the most useful translational insights are likely to emerge from coordinated, bidirectional study designs. Human work should inform the preclinical work and vice versa.

## 6. Conclusions

There is a major unmet need for a better understanding of major depressive disorder. While the tasks reviewed here vary in their degree of cross-species validation, the directly translatable behavioral tests described above provide a promising path to bridge human and rodent research on depression-related constructs. Continued efforts to standardize paradigms, expand multi-domain assessment, incorporate mechanistic modeling, and align experimental designs across species will be essential for realizing their full potential in basic neuroscience and the development of more effective treatments for mood disorders.

## Data Availability

No new data were created or analyzed in this study. Data sharing is not applicable to this article.

## Supplementary Materials

The following supporting information can be downloaded at: https://www.mdpi.com/article/10.3390/biology15090667/s1, Table S1: Summary of translatable human behavioral tests used by the studies included in the review; Table S2: Summary of translatable rodent behavioral tests used by the studies included in the review.

## Abbreviations

The following abbreviations are used in this manuscript:

ABT	Affective Bias Test
BDI	Beck Depression Inventory
CANTAB	Cambridge Neuropsychological Test Automated Battery
CEMT	Cognitive Effort Motivation Task
CMS	Chronic Mild Stress
CPT	Continuous Performance Test
CSDS	Chronic Social Defeat Stress
DA	Dopamine
dPAL	different Paired-Associates Learning
DSM	Diagnostic and Statistical Manual of Mental Disorders
ECT	Electroconvulsive Therapy
EDT	Effort Discounting Task
EEfRT	Effort-Expenditure for Rewards Task
FR	Fixed-Ratio
JBT	Judgment Bias Test
JORT	Joystick-Operated Runway Task
MDD	Major Depressive Disorder
mABT	modified Affective Bias Test
NFS	Negative Feedback Sensitivity
NIH RDoC	National Institute of Mental Health Research Domain Criteria
PAL	Paired-Associates Learning
PHQ	Patient Health Questionnaire
PRISMA	Preferred Reporting Items for Systematic Reviews and Meta-Analyses
PRL	Probabilistic Reversal Learning
PRT	Probabilistic Reward Task
rCET	rodent Cognitive Effort Task
rCPT	rodent Continuous Performance Test
RT	Reaction Time
STAI	State-Trait Anxiety Inventory
STT	Sweet Taste Test
VAS	Visual Analog Scale
WCST	Wisconsin Card Sorting Test
