# Supplementary material for: A Review of Translational Behavioral Assays in Depression Research
Source: Biology (Basel). 2026 Apr 23;15(9):667. doi: 10.3390/biology15090667 (PMC13162779; doi:10.3390/biology15090667)
Supplement: Supplementary file 1 [file biology-15-00667-s001.zip › Table S1 final.pdf]

Table S1 – Translatable Human Paradigms

Task	Paradigm Description	Primary Constructs	Key Findings	References	Advantages	Limitations
**Judgement Bias Test (JBT)**	Ambiguous tone discrimination with two reference tones predicting different outcomes (large vs small monetary reward, or reward vs aversive sound). Ambiguous mid-tones reinforced probabilistically; reduced tendency to choose the better outcome indicates negative bias.	Negative interpretive bias under ambiguity; affective bias.	Avoidance at most ambiguous tones correlates with anxiety even after controlling for depressive symptoms.<br>Subjects with depressed mood/elevated anxiety showed reduced high-reward choices and lower drift rate for ambiguous tones.	[28]<br>[29]	Directly measures cognitive bias under ambiguity; nearly directly analogous to rodent version; cues can be manipulated easily.	Not validated in patients with depression; human and rodent rewards are different.
**Continuous Performance Test (CPT)**	Vigil CPT: rapid serial letter presentation; respond only to target sequence (“A” followed by “K”). Measures hits, false alarms, RT, and RT variability.	Sustained attention / vigilance; intra-individual RT variability.	MDD and bipolar depression patients show increased RT variability despite similar accuracy to controls.	[31]	Well-established measure of sustained attention; highly similar task structure to rodent paradigm.	Limited evidence linking task performance to core depressive symptoms.
**Probabilistic Reward Test (PRT)**	Two-alternative discrimination (e.g., long vs short mouth on a face) with asymmetric reinforcement (rich vs lean stimulus). Response bias (log b) toward the rich stimulus measures reward responsiveness.	Reward responsiveness; probabilistic reward learning; anhedonia.	Higher BDI scores associated with blunted response bias despite intact discrimination, indicating reduced hedonic capacity.	[38]	Strong construct validity; validated in MDD patients; directly analogous to rodent paradigm.	Requires a different rich:lean reinforcement ratio than rodents.
**Probabilistic Reversal Learning (PRL)**	Two stimuli with asymmetric reward probability (typically 80:20); contingencies reverse after criterion. Win-stay and lose-shift behavior yields reward/punishment sensitivity metrics including NFS.	Cognitive flexibility; feedback sensitivity; negative valence / cognitive control interaction.	MDD patients show intact acquisition and reversal but significantly elevated NFS (excess lose-shift after probabilistic punishment) vs controls.	[43]	Validated in MDD patients; very similar to rodent version.	NFS differs from rodents; MDD patients are unimpaired in reversal learning.
**EEfRT / JORT**	EEfRT: trial-by-trial choice between low-effort/low-reward vs high-effort/high-reward with varying magnitude and probability. JORT: joystick force to “chase” a target.	Effort-based decision-making; physical effort cost; psychomotor fatigue; motivational anergia.	Higher state/trait anhedonia and depressive symptoms predict fewer high-effort choices, especially at low reward probability.<br>JORT did not differentiate high vs low anhedonia groups.	[49,50]<br>[49]	Directly measures motivational component of anhedonia; button presses analogous to rodent lever presses.	Not yet validated in MDD patients.
**Paired Associates**	CANTAB PAL: patterns appear in boxes at different	Visuospatial associative	Mixed/manic bipolar and MDD patients make more errors vs	[51,52,54]	CANTAB standardization	Does not assay core depressive symptoms

Task	Paradigm Description	Primary Constructs	Key Findings	References	Advantages	Limitations
**Learning (PAL)**	locations; after delay, patterns must be placed in original locations. Increasing set size (2, 3, 6, 8).	memory; learning and memory; cognitive impairment in mood disorders.	controls; performance improves in remission but remains impaired.		ensures consistency; near-identical stimulus presentation and outcome metrics across species; validated in MDD patients.	(anhedonia/depressed mood).
**Cognitive Effort Motivation Task (CEMT)**	Trial-by-trial choice between low-load/low-reward (1 location, 1 point) vs higher memory loads for more points (2–5 locations, 2–8 points). Performance contingent on ≥4/5 correct probes.	Cognitive effort cost; willingness to engage in cognitively demanding tasks.	MDD patients report higher cognitive demand and choose high-effort options less frequently across reward/effort levels, despite intact accuracy and decision times.	[58]	Separates cognitive effort from physical effort; validated in MDD patients.	Not directly analogous to rodent version.
**Wisconsin Card Sorting Test (WCST)**	Sort response cards by hidden rule (color, shape, number); rule changes after 10 consecutive correct. Measures categories completed, trials to criterion, perseverative/non-perseverative errors.	Cognitive flexibility; set-shifting; rule maintenance; executive dysfunction.	Dysphoric subjects complete fewer categories, require more trials, and commit more errors than controls.	[60]	Well-established measure of cognitive flexibility and set-shifting.	Limited specificity to mood disorders; not validated in MDD patients.
**Sweet Taste Test (STT)**	Taste ascending sucrose concentrations (0–40%), hold in mouth then spit and rinse; determine detection threshold and rate pleasantness across concentrations.	Sensory hedonic capacity; gustatory detection threshold; anhedonia.	Depressed patients show higher sweet taste perception thresholds and altered pleasure/displeasure ratings; higher physical anhedonia relates to lower sucrose pleasantness.	[61]	Strong face and construct validity; validated in MDD patients.	Influenced by metabolic state and taste preferences.
**Affective Bias Test (ABT)**	Go/no-go tasks with emotionally valenced words (positive vs negative) as target/distractor; rule reversals after blocks. Measures RTs, commission errors, and omissions.	Mood-congruent affective bias; emotional go/no-go; affective processing	MDD patients show impaired performance when positive words are targets, indicating negative bias.	[65,66]	Captures mood-congruent processing biases; validated in MDD patients.	Not directly analogous to rodent version.
			Manic patients show opposite pattern with positive bias.	[65,66]		
			Suicidal individuals show faster responses and more commission errors to negative words, indicating heightened mood-congruent bias.	[67]		

Abbreviations: 2CRS = Two-Choice Rule Switching Task; ABT = Affective Bias Test; ACC = Anterior Cingulate Cortex; BDI = Beck Depression Inventory; CANTAB = Cambridge Neuropsychological Test Automated Battery; CEMT = Cognitive Effort Motivation Task; CPT = Continuous Performance Test; dPAL = Delayed Paired Associates Learning; EDT = Effort Discounting Task; EEfRT = Effort-Expenditure for Rewards Task; FR = Fixed Ratio; JBT = Judgement Bias Task; JORT = Joystick-Operated Runway Task; mABT = Modified Affective Bias Test; MDD = Major Depressive Disorder; NFS = Negative Feedback Sensitivity; PAL = Paired Associates Learning; PROG = Progressive Ratio; PRL = Probabilistic Reversal Learning; PRT = Probabilistic Reward Task; rCET = Rodent Cognitive Effort Task; RT = Reaction Time; STT = Sweet Taste Test; VR = Virtual Reality; WCST = Wisconsin Card Sorting Test.
