# Supplementary material for: A Review of Translational Behavioral Assays in Depression Research
Source: Biology (Basel). 2026 Apr 23;15(9):667. doi: 10.3390/biology15090667 (PMC13162779; doi:10.3390/biology15090667)
Supplement: Supplementary file 1 [file biology-15-00667-s001.zip › Table S2 final.pdf]

Table S2 – Translatable Rodent Tests

Task	Paradigm Description	Primary Constructs	Key Finding	References	Advantages	Limitations
**Judgement Bias Test (JBT)**	Spatial JBT: radial arm maze with "positive" (home cage) and "negative" (air puff) arms; ambiguous probe arms intermediate between them. Auditory JBT: reward vs punishment tones with ambiguous mid-tone; lever choice unreinforced.	Negative interpretive bias under ambiguity; affective bias.	Chronic social stress shifts choices toward punishment-avoidance on ambiguous tones.	[27]	Directly measures cognitive bias under ambiguity; nearly directly analogous to human version; cues can be manipulated easily; validated in rodent stress model.	Human and rodent rewards are different.
			Pessimistic training history slows approach to ambiguous spatial arms, indicating negative bias.	[26]		
**Continuous Performance Test (CPT)**	Touchscreen CPT: rats nose-poke to a single rewarded stimulus while withholding responses to four non-signal stimuli. Measures hits, misses, false alarms, correct rejections, RT, and signal detection metrics.	Sustained attention / vigilance; signal detection sensitivity.	Stimulant-like drugs increase hits, false alarms, and response bias; non-stimulants reduce these measures, validating sensitivity to attention-modulating compounds.	[30]	Well-established measure of sustained attention; highly similar task structure to human paradigm.	Limited evidence linking task performance to core depressive symptoms.
**Probabilistic Reward Test (PRT)**	Touchscreen or auditory discrimination with asymmetric reinforcement; correct choices rewarded with food/sucrose.	Reward responsiveness; probabilistic reward learning; anhedonia.	Rats and mice acquire the task and show response bias to the rich stimulus.	[32-36]	Strong construct validity; validated in rodent stress models; directly analogous to human paradigm.	Requires a different rich:lean reinforcement ratio than humans.
			~60:20 rich:lean contingency yields log b values similar to humans (~0.2–0.3); stress or dopaminergic manipulations modulate bias.	[35,36]		
**Probabilistic Reversal Learning (PRL)**	Touchscreen or hole-based two-choice tasks with probabilistic reinforcement; reward probabilities manipulated (80:20 and 90:10) across rat and mouse studies.	Cognitive flexibility; feedback sensitivity; punishment sensitivity.	Rats and mice show much higher NFS than humans at 80:20; raising correct reward probability to 90% reduces NFS in mice but remains higher than typical human controls, highlighting species differences in punishment sensitivity.	[39-41]	Very similar to human version.	Not validated in rodent stress models; NFS differs from humans.

Task	Paradigm Description	Primary Constructs	Key Finding	References	Advantages	Limitations
**Effort-Based Choice Tasks (FR/chow, T-maze, Touchscreen)**	FR/chow and PROG/chow: lever pressing for preferred food with freely available chow. T-maze barrier choice: climb barrier for high reward vs no barrier for low reward. Touchscreen FR/chow: rearing and pressing for milkshake vs free pellets.	Effort-based decision-making; physical effort cost; psychomotor fatigue; motivational anergia.	Dopamine antagonists/depletion and stress shift choice away from high-effort/high-reward options toward low-effort chow; antidepressant-like manipulations restore effortful choice in some paradigms.	[44-48]	Directly measures motivational component of anhedonia; lever presses analogous to human button presses.	Multiple different protocols for rodent version; not validated in rodent stress models.
**Delayed Paired Associates Learning (dPAL)**	Touchscreen dPAL: three visual symbols each associated with a specific window; rat must touch the symbol in its correct location to receive food reward.	Visuospatial associative memory; stress-sensitive cognitive impairment in mood disorders.	After chronic mild stress, stress-susceptible rats require more trials to reach criterion vs controls, while resilient animals perform similarly to controls.	[53]	Near-identical stimulus presentation and outcome metrics across species; validated in rodent stress models.	Does not assay core human depressive symptoms (anhedonia, depressed mood).
**Rodent Cognitive Effort Task (rCET)**	Five-hole operant chamber; rats choose low-effort/low-reward lever (long stimulus, 1 pellet) vs high-effort/high-reward lever (brief stimulus, 2 pellets).	Cognitive effort cost; effort-reward trade-offs; frontal circuit involvement.	ACC inactivation reduces high-effort choice and increases premature responses/omissions, implicating medial frontal circuits.<br><br>Dopamine antagonism selectively reduces high-effort physical choices but not cognitive effort in rCET.	[55-56]<br><br>[57]	Uniquely isolates cognitive effort from physical effort.	Not directly analogous to rodent version; not validated in rodent stress models.
**Two-Choice Rule Switching (2CRS)**	Visual two-choice rule-switch task in head-fixed VR: mice choose between stimuli differing in pattern and size; hidden rules switch after achieving criterion performance.	Cognitive flexibility; set-shifting; rule maintenance	Following rule changes, mice show increased trials to criterion, slower responses on conflicting trials, and primarily regressive errors (failure to maintain new rule).	[59]	Captures error types analogous to human WCST.	Head-fixed paradigm is less naturalistic and limits validity; not validated in rodent stress models.
**Brief-Access Taste Test**	Stressed and control mice given 5 s access to sucrose and other tastants via lick spout; responses quantified	Sensory hedonic capacity; gustatory	Chronic social defeat stress selectively reduces sensitivity to sweet and umami tastes but not salty, sour, or bitter.	[62]	Strong face and construct validity; validated in rodent stress models.	Does not measure sweet taste detection threshold *per* se; requires a gustometer.

Task	Paradigm Description	Primary Constructs	Key Finding	References	Advantages	Limitations
	as licks of tastant minus licks of water.	sensitivity; anhedonia.				
**Affective Bias Test (ABT / mABT)**	ABT: rats learn substrate–reward associations under neutral vs affective manipulations; later choice reveals bias. mABT: high- vs low-value reward substrates; chronic manipulations modulate bias.	Mood-congruent affective bias; affective learning and memory	Acute antidepressants induce positive bias toward drug-paired substrate.<br>Anxiogenic/stress manipulations induce negative bias.<br>Chronic pro-depressant manipulations blunt bias toward high-value reward; chronic antidepressant treatments maintain/enhance it.	[63,64]<br>[63,64]<br>[64]	Validated in rodent stress models.	Not directly analogous to human version.

Abbreviations: 2CRS = Two-Choice Rule Switching Task; ABT = Affective Bias Test; ACC = Anterior Cingulate Cortex; BDI = Beck Depression Inventory; CANTAB = Cambridge Neuropsychological Test Automated Battery; CEMT = Cognitive Effort Motivation Task; CPT = Continuous Performance Test; dPAL = Delayed Paired Associates Learning; EDT = Effort Discounting Task; EEfRT = Effort-Expenditure for Rewards Task; FR = Fixed Ratio; JBT = Judgement Bias Task; JORT = Joystick-Operated Runway Task; mABT = Modified Affective Bias Test; MDD = Major Depressive Disorder; NFS = Negative Feedback Sensitivity; PAL = Paired Associates Learning; PROG = Progressive Ratio; PRL = Probabilistic Reversal Learning; PRT = Probabilistic Reward Task; rCET = Rodent Cognitive Effort Task; RT = Reaction Time; STT = Sweet Taste Test; VR = Virtual Reality; WCST = Wisconsin Card Sorting Test.
